# Editorial: New insight into the diagnosis and treatment of age-related macular degeneration

**DOI:** 10.3389/fmed.2022.993349

**Published:** 2022-08-04

**Authors:** Andrea Servillo, Riccardo Sacconi, Giuseppe Querques

**Affiliations:** ^1^Ophtalmology Unit, Division of Head and Neck, IRCSS Ospedale San Raffaele, Milan, Italy; ^2^School of Medicine, Vita-Salute San Raffaele University, Milan, Italy

**Keywords:** age-related macula degeneration, anti-vascular endothelial growth factor, neovascularization, treatment, diagnosis

Age-related macular degeneration (AMD) is a leading cause of central visual loss in the developed world. During the last few years, the advent of intravitreal injections of anti-VEGF drugs has revolutionized the treatment of neovascular AMD (nAMD).

However, deterioration of visual acuity and disease persistence are observed in about 25% of treated patients (1). Numerous causes can lead to therapy failure, and a better knowledge of AMD pathogenetic mechanisms is required to expand the available therapies.

In this direction, Gu et al. showed that dysregulation of monocyte phagocytosis may occur in the early stages of AMD when debris accumulates, producing drusen, reticular pseudo drusen (RPD), and deposits within BM. In addition, they observed that defective phagocytosis could be reversed, in part, by glatiramer acetate, suggesting a potential novel intervention for the earlier stages of AMD (Gu et al.). Coronado et al. evaluated the protein profiles of the aqueous humor of three groups of patients to obtain new information on AMD pathophysiology: (1) nAMD patients with good response to therapy, (2) nAMD patients with anti-VEGF resistance, (3) control patients without signs of retinopathy. They found 39 proteins as potential disease effectors in lipid metabolism, oxidative stress, complement system, inflammatory pathways, and angiogenesis (Coronado et al.). This study contributed identifying promising biomarkers in nAMD to discover new possible target treatments or to predict treatment response.

Regarding the latter point, Hoffmann and Hatz evaluated the efficacy and outcome predicting factors in intravitreal anti-VEGF therapy following a treat-and-extend regimen (T&E) with exit strategy in treatment-naïve patients for up to 8 years. They concluded that baseline characteristics such as the integrity of external limiting membrane (ELM) or the absence of pigment epithelial detachment (PED) predicted the probability of reaching exit criteria and may be helpful in adapting treatment strategies (Hoffmann and Hatz). Indeed, treatment failure depends not only on the limited therapeutic choices at our disposal but also on an incorrect patient's selection. Macular neovascularization (MNV) associated lesions such as fibrovascular PED may contribute to treatment failure. Furthermore, some MNV subtypes, such as polypoidal choroidal vasculopathy (PCV) exhibit a more aggressive behavior and tendency to relapse.

Due to this, the research is focusing on PCV lesions. Wang et al. helped clarify the comprehensive research progress in the study of PCV. They collected all the publications in this field from 2001 to 2020, and analyzed trends within them, founding that PCV is becoming an increasingly relevant topic of research. Their results can help clinicians understand the history of PCV research and make better clinical decisions (Wang et al.).

Furthermore, Jia et al. evaluated the efficacy and safety of the T&E and PRN regimen of conbercept in Chinese patients with nAMD, specifically to demonstrate the non-inferiority of the T&E regimen as compared with the PRN regimen. However, the T&E regimen with conbercept did not meet the primary objective (Jia et al.). Contrairly, recent studies in caucasian patients found that treatment with the T&E regimen was non-inferior to the PRN regimen (2, 3). The authors attribute this disparity to a variety of factors, including a higher prevalence of PCV in Asian patients than in caucasians. As previously stated, PCV exhibits more aggressive behavior and may necessitate a more aggressive treatment regimen.

Thanks to the published articles, this Research Topic on new insight into the diagnosis and treatment of AMD may impact the current and future knowledge on pathogenesis and treatment of AMD patients. Patient's selection, treatment regimen, and potential marker for treatment response can help the future management of this pathology. For this reason, we truly believe that the finding showed in this Research Topic may improve the knowledge on diagnosis and treatment of AMD.

## Author contributions

AS, RS, and GQ wrote the paper. All authors contributed to the article and approved the submitted version.

## Conflict of interest

RS has the following disclosures: Allergan Inc., Bayer Shering-Pharma, Medivis, Novartis, Zeiss. GQ has the following disclosures: Alimera Sciences, Allergan Inc., Amgen, Bayer Shering-Pharma, Heidelberg, KBH, LEH Pharma, Lumithera, Novartis, Sandoz, Sifi, Sooft-Fidea, Zeiss. The remaining author declares that the research was conducted in the absence of any commercial or financial relationships that could be construed as a potential conflict of interest.

## Publisher's note

All claims expressed in this article are solely those of the authors and do not necessarily represent those of their affiliated organizations, or those of the publisher, the editors and the reviewers. Any product that may be evaluated in this article, or claim that may be made by its manufacturer, is not guaranteed or endorsed by the publisher.
